# Hemodynamic Instability Secondary to Inferior Vena Cava Compression: A Rare Complication of Massive Walled-off Pancreatic Necrosis

**DOI:** 10.14309/crj.0000000000000269

**Published:** 2019-10-24

**Authors:** Tanvi Goyal, Toseef Javaid, Anirudh Goyal, Zubair Khan

**Affiliations:** 1 Department of Internal Medicine, University of Toledo College of Medicine, Toledo, OH; 2 Department of Gastroenterology, University of Toledo College of Medicine, Toledo, OH; 3 Department of Internal Medicine, Rutgers New Jersey Medical School, Newark, NJ

## Abstract

Necrosis developing 4 weeks after the initial acute pancreatitis attack is known as walled-off pancreatic necrosis (WOPN). Complications of WOPN include spontaneous rupture into the peritoneal cavity or hollow viscus obstruction by compression of surrounding structures, including the colon, stomach, duodenum, and common bile duct. There have also been cases of pseudocyst rupture into blood vessels. This case report is unique in that it highlights a patient with inferior vena cava compression leading to hemodynamic instability due to the mass effect of WOPN and has not been previously reported.

## INTRODUCTION

Severe acute pancreatitis associated with necrosis leads to significant morbidity and mortality.^1^ Necrosis seen within the first 4 weeks of pancreatitis is defined as an acute necrotic collection, whereas necrosis developing 4 weeks after the initial attack is known as walled-off pancreatic necrosis (WOPN), in which the fluid collection is delineated by a fibrotic and inflammatory wall.^2^ The mortality rate of WOPN is, however, less than that of infected pancreatic necrosis (pancreatic abscess). Most uncomplicated acute pancreatic fluid collections are managed conservatively unless symptomatic or infected.^2^ In these more severe cases, minimally invasive approaches, including endoscopic drainage of WOPN, should be considered. Complications of WOPN include spontaneous rupture into the peritoneal cavity or hollow viscus obstruction by compression of surrounding structures, including the colon, stomach, duodenum, and common bile duct.^3^ There have also been cases of pseudocyst rupture into blood vessels.^4^ This case is unique in that it highlights a patient who developed hemodynamic instability from inferior vena cava (IVC) compression secondary to mass effect from WOPN, necessitating further intervention.

## CASE REPORT

A 56-year-old man with a history of alcoholism presented to a local hospital for right lower quadrant abdominal pain and elevated serum amylase. His lipase level was 1,130 U/L. No jaundice or signs of ascending cholangitis were identified. The initial computed tomography (CT) scan with intravenous contrast showed moderate amounts of pancreatic necrosis, mainly on the right side of the abdomen. To delineate the integrity of the pancreatic duct, magnetic resonance cholangiopancreatography was performed. Magnetic resonance cholangiopancreatography showed a dilated main pancreatic duct proximally toward the head and neck of the pancreas with tapering distally toward the body and tail and a hypointense filling defect in the distal main pancreatic duct just before the insertion into the ampulla.

**Figure 1.**
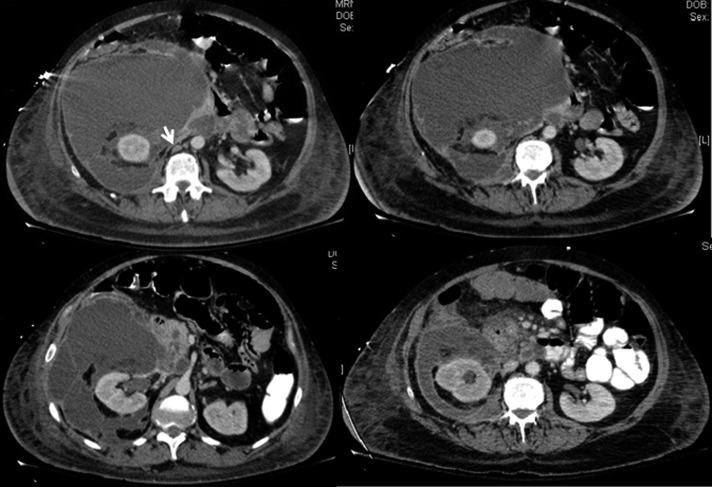
Abdominal computed tomography showing large amounts of retroperitoneal fluid involving the right perinephric spaces with kidney displacement. The arrow points to compression of the inferior vena cava secondary to mass effect from walled-off pancreatic necrosis.

The patient was treated conservatively for acute necrotizing pancreatitis (ANP), but his abdominal pain did not improve significantly over the subsequent 10 days. A repeat CT scan was performed, which revealed increasing amounts of retroperitoneal fluid mainly to the right of the midline involving the right perinephric and retronephric spaces, causing displacement of the right kidney and poor delineation of the IVC secondary to a mass effect (Figure 1). A calcific density in the distal pancreatic head suspicious for an obstructing common bile duct stone was also noted. The patient was subsequently transferred to our facility for endoscopic retrograde cholangiopancreatography. However, given that the patient was hemodynamically stable, a decision was made to perform a delayed endoscopic retrograde cholangiopancreatography in 4–6 weeks.

Over the next 2 weeks, the patient developed progressive abdominal distention, followed by sudden-onset shortness of breath, and he became hemodynamically unstable. The patient was transferred to the intensive care unit, and another CT scan was performed which showed massive WOPN, IVC collapse, and a right hydroureter. An emergent CT-guided percutaneous drainage was performed, which resulted in improvement of the respiratory distress and hemodynamics over the next 48 hours. The patient was determined to have hemodynamic instability secondary to impaired venous return and respiratory distress secondary to decreased total lung capacity, leading to compressive atelectasis, both of which markedly improved as the fluid was drained. The patient was then discharged 3 days later to a long-term acute care facility.

## DISCUSSION

The incidence of pancreatitis in the United States is approximately 185,000 cases per year. ANP is reported to occur in approximately 20% of all episodes of pancreatitis. WOPN, formerly known as a pancreatic abscess, is a late complication of severe acute pancreatitis, occurring 4 weeks after the episode in 1-9% of cases.^1^ The term WOPN was first used by Connor et al in 2005 and was subsequently officially established in 2006 by the American Gastroenterological Association.^3^ Pancreatitis with an unexpectedly long course, unresponsiveness to medical therapy, hemodynamic instability, and fluid on CT indicate the possibility of necrosis and later WOPN development. Medical treatment is generally supportive, and antibiotics such as imipenem can be administered to patients with bacteremia. The ultimate treatment of choice for WOPN, however, would consist of drainage to avoid increased morbidity and mortality.

ANP, depending on the degree of necrosis and surgical debridement, can have great implications on the morbidity of the patient, including insufficient endocrine and exocrine functioning of the pancreas to significantly affect the patient's quality of life.^1^ Severe complications of WOPN include fistula formation, recurrent pancreatitis, rupture into the peritoneal cavity, and hollow viscus obstruction by compression of surrounding structures, including the colon, stomach, duodenum and common bile duct.^3^ If left untreated, WOPN can progress to sepsis and death.

An important consideration in WOPN is vascular complications. There have been cases of pseudocyst rupture into blood vessels, including the portal vein, leading to thrombosis and subsequent lysis of the thrombus by pancreatic enzymes. Communication of the pseudocyst and portal venous system can be seen on imaging. Similarly, a case report by Ramachandran et al reported a case of WOPN rupturing into the IVC leading to its thrombosis.^4^ Although cases of IVC thrombosis in necrotizing pancreatitis have been seen, no cases of WOPN directly compressing the IVC and leading to hemodynamic instability have been reported. This emphasizes the rarity of this complication and the critical role of imaging and decompression to relieve the mass effect before progression to hemodynamic instability and shock.

Sepsis is the primary concern in patients presenting with hypotension and history of WOPN. This case highlights that, although rare, impaired venous return secondary to a mass effect due to a large pancreatic collection should be considered in the differential diagnosis for all patients with a history of recent pancreatitis presenting with shock. In addition, this case demonstrates that early endoscopic or CT-guided drainage may be of benefit in select cases of massive pancreatic fluid collections in a small subgroup of patients who have radiologic evidence of compression of vascular structures or vital organs before symptoms of clinical deterioration develop. Early detection and intervention measures should be taken in cases of WOPN to decrease mortality and morbidity from this disease and its complications.

## DISCLOSURES

Author contributions: All the authors contributed equally to this manuscript. T. Goyal is the article guarantor.

Financial disclosure: None to report.

Informed consent was obtained for this case report.

Previous presentation: This case was presented at the American College of Gastroenterology Annual Scientific Meeting; October 16–21 2015; Honolulu, Hawaii.
